# Chemical Optimization of Temporin A–L Peptides: From Native Isoforms to Enhanced Antimicrobial Analogs

**DOI:** 10.1002/cmdc.70397

**Published:** 2026-07-31

**Authors:** Ida Boccino, Paolo Grieco, Francesco Merlino

**Affiliations:** ^1^ Department of Pharmacy University of Naples Federico II Naples Italy

**Keywords:** antimicrobial peptides (AMPs), membrane‐active peptides, sequence modifications, structure–activity relationship (SAR), temporins

## Abstract

Temporins A–L, a family of short antimicrobial peptides isolated from the skin secretions of *Rana temporaria*, have emerged as promising scaffolds for the development of novel anti‐infective agents. However, their therapeutic translation is limited by issues such as modest selectivity, susceptibility to proteolytic degradation, and variable potency. In this review, we provide an overview of the medicinal chemistry efforts devoted to the optimization of this class of AMPs through systematic chemical modifications. Strategies including backbone alterations (e.g., D‐amino acid incorporation and retro‐inverso design), side chain modulation, charge tuning, hydrophobicity adjustment, and the introduction of noncanonical residues are discussed in relation to their impact on antimicrobial activity and selectivity. Particular attention is given to conformationally constrained analogs, such as cyclic, stapled, and bicyclic derivatives, which often display enhanced stability and bioactivity. By integrating available data, we delineated key structure–activity relationships governing temporin function and identified emerging design principles for the development of improved analogs. Finally, current challenges and future perspectives for the advancement of temporin‐based therapeutics in medicinal chemistry are outlined.

## Graphical abstract

Temporins, among antimicrobial peptides, are short frog‐derived molecules with potent antibacterial activity. This review highlights chemical optimization strategies applied to temporins A–L, showing how sequence tuning modulates structure, stability, and selectivity, enabling improved activity against resistant pathogens.

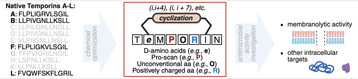

## 1 Introduction

**FIGURE 1:**
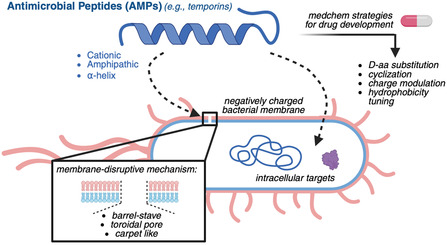
Schematic representation of the main structural features and antimicrobial mechanisms of AMPs, with particular reference to temporins.

The increasing incidence of bacterial infections, together with the excessive and inappropriate use of existing antibiotics, has accelerated the emergence of bacterial tolerance and resistance [1]. This phenomenon represents a serious global threat to human health and has been consistently highlighted by the World Health Organization (WHO), which recognizes antimicrobial resistance as *“one of the top global public health and development threats”* [2]. Despite the continuous rise of resistant bacterial strains, the development of truly innovative antibacterial agents remains limited [3, 4, 5, 6, 7]. Although nearly 230 antibacterial agents were in preclinical development as of 2025 [8], several are derivatives of existing antibiotic classes, offering limited mechanistic novelty. These considerations underscore the urgent need for alternative therapeutic strategies that can overcome conventional resistance mechanisms while ensuring sustained efficacy. In this context, antimicrobial peptides (AMPs) have emerged as promising candidates [9, 10, 11, 12, 13, 14, 15]. Originally identified as key components of innate immunity across a wide range of organisms, including animals, amphibians, and plants, AMPs typically exhibit broad‐spectrum activity and a lower propensity to induce resistance compared to traditional antibiotics. Their activity is primarily associated with interactions with bacterial membranes, although immunomodulatory effects and additional intracellular mechanisms have also been reported [16, 17, 18, 19, 20]. Despite their structural diversity, AMPs share common physicochemical features, such as a net positive charge, amphipathicity, and a tendency to adopt α‐helical conformations upon membrane interaction. These properties are central to their biological activity and can be tuned rationally. In particular, electrostatic interactions with negatively charged membrane components, combined with hydrophobic insertion into the lipid bilayer, drive membrane destabilization and ultimately cell death [21, 22, 23, 24, 25, 26, 27, 28, 29, 30]. Depending on their sequence and structure, AMPs can disrupt membranes through different modes of interaction (e.g., barrel‐stave, carpet, or toroidal models), or, in some cases, translocate across the membrane and target intracellular processes such as nucleic acid or protein synthesis (Figure 1) [31, 32].

**FIGURE 2:**
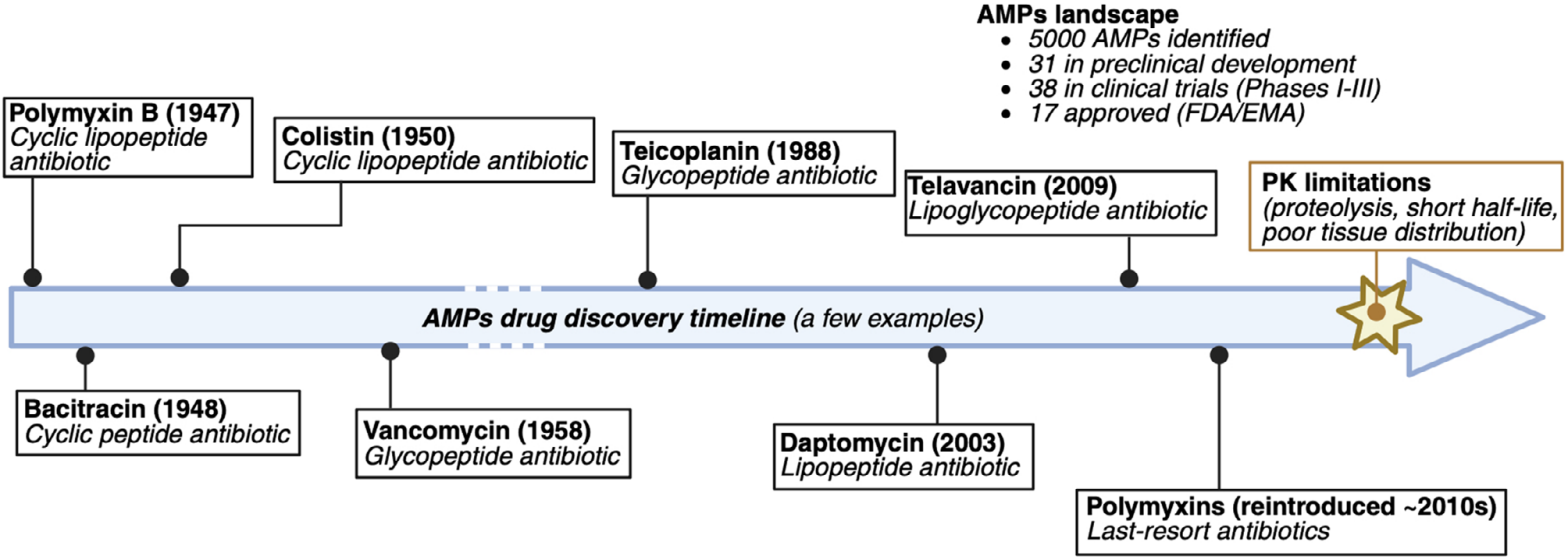
Overview of the antimicrobial peptide therapeutic landscape and timeline of selected AMP‐derived antibiotics.

A limited number of peptide‐based anti‐infective agents, mostly derived from or inspired by natural AMPs, such as daptomycin, vancomycin, and related glyco‐ and lipo‐peptides, have reached clinical use, although they represent only a small fraction of the currently approved peptide therapeutics. Overall, while more than 80 peptide‐based drugs are currently approved for clinical use across different therapeutic areas, only a few are employed as anti‐infective agents (Figure 2) [33]. Regarding AMPs, over 5000 have been described, yet only 31 have reached preclinical trials, 38 are in phases I–III, and 17 have been FDA and/or EMA‐approved and are currently on the market (Table 1) [34]. However, their therapeutic application is still hindered by significant limitations, including poor proteolytic stability and cytotoxicity, often observed at concentrations close to those required for antimicrobial activity. To address these challenges, a wide range of rational design and chemical modification strategies have been developed to improve their stability, selectivity, and overall drug‐like properties [35, 36, 37, 38, 39, 40, 41, 42]. In this framework, temporin peptides, and particularly the temporin A–L family, represent a valuable model for exploring structure–activity relationships (SAR) and possibly guiding the development of optimized AMP‐based therapeutics.

**TABLE 1:** FDA‐ and/or EMA‐approved AMPs currently on the market: sequence, source, medical use, and molecular target.

Name	Sequence	Source	Activity: medical use	Target
Bacitracin^a^	LeIKornIfHdN	*Bacillus licheniformis*	Antibacterial: prevent pneumonia and empyema in infants, skin and eye infections	Cell wall
Dalbavacin (Xydalba)	*n.a.*	Semi‐synthetic	Antibacterial: acute bacterial skin infections, osteomyelitis and septic arthritis	Cell wall
Daptomycin (Cubicin)	Decanoyl‐WNTGornDaDGsED	*Streptomyces roseosporus*	Antibacterial: complicated skin infections and bloodstream infections (bacteremia)	Cell membrane
Enfuvirtide	YTSLIHSLEESQNQQEKNEQELLELDKWASLWNWF	Synthetic	Anti‐Human Immunodeficiency Virus (HIV)	Fusion protein gp41
Gramicidin D^a^	VGALAVVVWLWLWLW‐ethanolamine	*Bacillus brevis*	Antibacterial: skin lesions and eye infections	Cell membrane
Gramicidin S^a^	[LfPVOrnLfPVOrn]^b^	*Bacillus brevis*	Antibacterial: spermicide against bacteria and fungi; genital ulcers	Cell membrane
Obiltoxaximab^a^	*n.a.*	Monoclonal antibody	Antibacterial: inhalational anthrax	Antitoxin
Oritavancin^a^	*n.a.*	Semi‐synthetic	Antibacterial: inhalational anthrax	Antitoxin
Obiltoxaximab^a^	*n.a.*	Monoclonal antibody	Antibacterial: acute bacterial skin infections	Cell wall
Palivizumab	*n.a.*	Monoclonal antibody	Antiviral: prevent serious lung infections caused by respiratory syncytial virus‐RSV	Blocking viral replication
Polymyxin B^a^	*n.a.*	*Bacillus polymyxa*	Antibacterial: infections of the urinary tract, meninges, and blood stream	Cell membrane
Polymyxin E	6‐mh‐DabTDab[DablLDabDabT]^c^	*Bacillus polymyxa*	Antibacterial: acute or chronic infections due to Gram‐negative bacilli	Cell membrane
Raxibacumab^a^	*n.a.*	Semi‐synthetic	Antibacterial: inhalational anthrax	Antitoxin
Telavancin	*n.a.*	Semi‐synthetic	Antibacterial: osteomyelitis and bacterial infections	Cell membrane
Tyrothricin (Vibativ)^a^	*n.a.*	*Brevibacillus parabrevis*	Antibacterial and Antifungal: infected skin and infected oropharyngeal mucous membranes	Cell membrane
Thymalfasin^a^	SDAAVDTSSEITTKDLKEKKEVVEEAEN	Synthetic	Antiviral: hepatitis B and C	Immuno‐modulator
Vancomycin	*n.a.*	*Amycolatopsis orientalis*	Antibacterial: septicemia, infective endocarditis, skin, bone and lower respiratory tract infections	Cell membrane

Abbreviation: n.a.: not available.

a Not authorized by the European Medicines Agency (EMA).

b Head‐to‐tail cyclization.

c Head‐to‐side chain cyclization.

## 2 Temporin Isoforms From *Rana temporaria*

**FIGURE 3:**
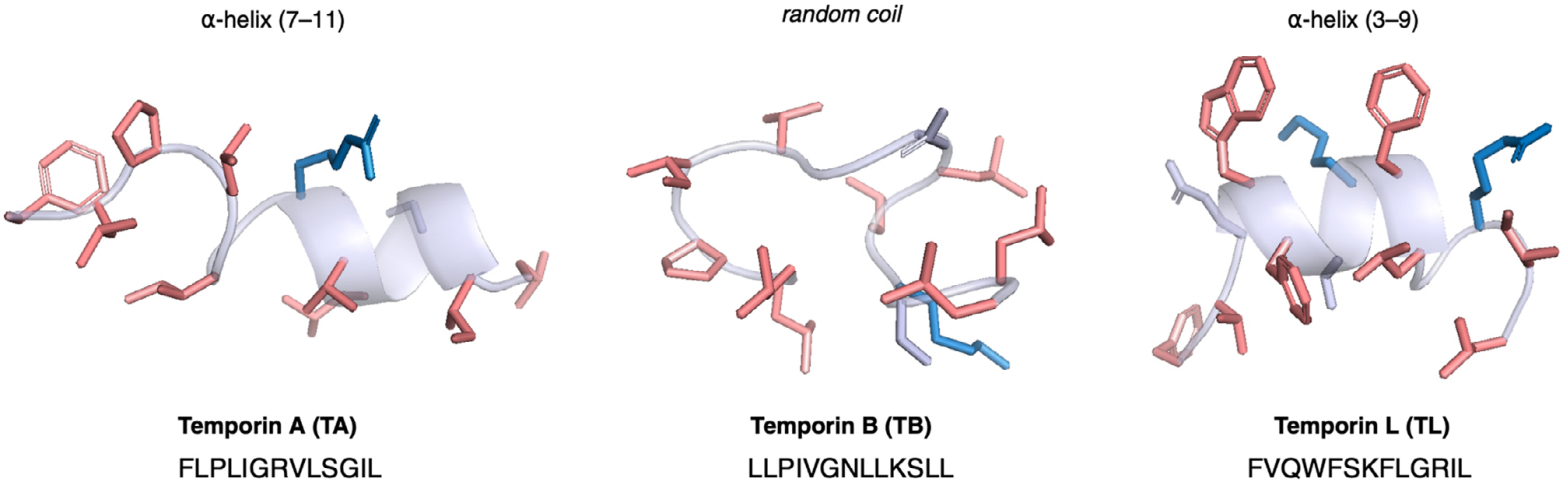
Representative three‐dimensional structures and amino acid sequences of selected temporin family peptides. Temporin A (TA) (PDB id: 2MAA) and temporin L (TL) (PDB id: 6GS5) adopt predominantly α‐helical conformations, spanning residues 7–11 and 3–9, respectively, whereas temporin B (TB) (PDB id: 6GIL) displays a mainly random coil structure [61, 62, 63]. Polar residues are shown in blue, nonpolar residues in salmon and the peptide backbone in light gray.

Temporins represent an attractive class of peptides for biological and pharmacological investigations due to their short length, relatively low synthesis cost, and broad‐spectrum antimicrobial activity [43, 44]. In addition to their direct antimicrobial effects, several temporins also possess chemotactic and immunomodulatory properties [45, 46, 47, 48, 49, 50, 51]. These peptides share common structural features, including a high content of hydrophobic residues, often accounting for ∼70% of the peptide sequence, and an amphipathic α‐helical conformation [52, 53]. Temporins A–L, which are isolated from the skin secretions of *Rana temporaria*, generally consist of 10 or 13 amino acids and display a relatively low net positive charge at neutral pH (ranging from 0 to +3), features that distinguish them from many other cationic AMPs. These temporins exhibit activity against a wide range of pathogens, including bacteria, fungi, viruses, and protozoa, with the exception of D and H isoforms, which show little or no activity under standard experimental conditions [54, 55, 56, 57, 58, 59]. The mechanism of action of temporins A–L is mainly associated with perturbation of the cytoplasmic membrane, although it some cases the activity is related to an intracellular mechanism [60]. Their activity strongly depends on their conformational adaptability, as temporins generally adopt α‐helical structures upon interaction with membrane‐mimicking environments while remaining more flexible in solution (Figure 3) [61, 64, 65, 66].

Temporin A (TA), first identified in 1996, is a 13‐mer peptide (FLPLIGRVLSGIL‐NH_2_) with a net positive charge of +2 [48]. TA exhibits activity against Gram‐positive bacteria, such as *Staphylococcus aureus* strains (MIC of 2.6–5.2 µM) and *Bacillus megaterium* (MIC value 1.2 µM), Gram‐negative bacteria, such as *Escherichia coli* strains (MIC value 11.9 µM) and clinically relevant strains such as methicillin‐resistant *S. aureus* (MRSA) strains (MIC values 0.9–2.7 µM) and vancomycin‐resistant *Enterococcus faecium* (VRE) strains (MIC values 5–21.5 µM), and *Enterococcus faecalis* (MIC value 10 µM), fungi and protozoa and has also been reported to display anticancer and insulinotropic activity [44, 48, 57]. TA can also act synergistically with conventional antibiotics and other temporins [67]. Its mechanism of action is primarily membrane‐based. In particular, at increasing peptide concentration, TA accumulates on the membrane surface, leading to peptide self‐assembly and membrane disruption through a carpet‐like mechanism [65, 68]. Structurally, TA is unordered in aqueous solution but adopts an α‐helical conformation in membrane‐mimicking environments, such as in the presence of cosolvents (e.g*.*, trifluoroethanol, TFE) or detergents (e.g., sodium dodecyl sulfate, SDS), consistent with its active form. In contrast, in the presence of dodecylphosphocholine (DPC) micelles, TA does not fold and is not able to penetrate the hydrophobic core, suggesting a rationale for its low hemolytic activity. Temporin B (TB) (LLPIVGNLLKSLL‐NH_2_), with a net charge of +2 [48], exhibits activity mainly against Gram‐positive bacteria, including *S. aureus* (MIC value 6 µM), *B. megaterium* (MIC value 2.8 µM) and *Streptococcus pyogenes* (MIC value 7 µM), as well as against fungi such as *Candida albicans*, and shows limited activity against Gram‐negative species, such as *Acinetobacter baumanii* (MIC values 32–64 µM) [48, 62]. Similar to TA, TB lacks a defined structure in aqueous solution but adopts an α‐helical conformation in membrane‐mimicking environments. Interestingly, in the presence of lipopolysaccharide (LPS) or whole bacterial cells, TB does not form a well‐defined structure, likely due to competing effects between membrane interaction and peptide self‐aggregation as suggested by NMR studies. These studies further indicate that LPS interaction is primarily mediated by hydrophobic and polar residues, whereas nonpolar residues promote peptide self‐aggregation, thereby limiting interaction with LPS micelles [63]. Temporin F (TF) (FLPLIGKVLSGIL‐NH_2_), with a net charge of +2 [48], has been reported to exhibit activity against selected bacterial species, including *E. coli* (MIC value 64 µM) while maintaining relatively low toxicity toward human red blood cells (less than 5% of cell lysis at 100 µmol/L) [69]. Temporin L (TL) (FVQWFSKFLGRIL‐NH_2_), with a net charge of +3 [48], is one of the most potent members of the family, displaying broad‐spectrum antimicrobial activity, against both Gram‐positive and Gram‐negative bacteria, including *Pseudomonas aeruginosa* strains (MIC values 24–48 µM) and *E. coli* (MIC values 6–12 µM) [70]. It also exhibits antibiofilm properties and antiviral activity. TL interacts strongly with lipid membranes, leading to their destabilization, likely through transient pore formation or localized disruptions. This activity is supported by its ability to adopt an α‐helical conformation in membrane‐mimicking environments [63]. In addition to membrane activity, TL has also been shown to exert intracellular effects in Gram‐negative bacteria. In *E. coli*, TL can interact with the cell division protein FtsZ, impairing its function and contributing to bacterial cell death [71, 72]. To date, among the *Rana temporaria* temporin A–L isoforms and derivatives discussed in this review, TL remains the only peptide for which an intracellular target has been experimentally validated. Despite its high antimicrobial potency, TL is accompanied by significant hemolytic activity and cytotoxicity toward mammalian cells, including cancer cell lines [55].

Temporin G exhibits activity against both Gram‐positive bacteria and has been primarily reported as synergistic agent, to enhance the activity of antibiotics such as tobramycin when used at sub‐inhibitory concentrations and to exert antiviral effects, particularly at early stages of viral replication [73, 74, 75, 76]. Other isoforms, including temporins C, E, and K, have been less extensively characterized. These peptides generally share the typical structural features of the temporin family but display only moderate antimicrobial activity. Temporins D and H are also poorly characterized and appear largely inactive under standard experimental conditions. Overall, detailed SAR and mechanistic insights for these isoforms remain limited.

## 3 Strategies for Temporins A–L Chemical Optimization: Linear Analogs

In light of the above considerations, efforts to elucidate SAR and to develop rational design strategies aimed at improving their pharmacological properties have primarily been predominantly focused on a subset of *Rana temporaria* temporins, namely temporins A, B, L, and, to a lesser extent, F, which combine measurable antimicrobial activity with sufficient structural and mechanistic characterization to support systematic chemical modification. Accordingly, the following sections will focus on these temporins, providing a comprehensive overview of the chemical and structural modifications reported to date, in order to identify key SAR trends that can guide the rational design of next‐generation antimicrobial agents (Table 2).

**TABLE 2:** Linear analogs of temporins A–L obtained by chemical optimization.

Peptide ID (#)	Sequence^a^	Activity	References
*Backbone modifications:*			
*Rev‐TA* (**1**)	LIGSLVRGILPLF	Reduced activity versus Gram‐positive strains; altered helical dipole	[44]
*D‐TA* (**2**)	*flpligrvlsgil*	Activity comparable to TA; increased proteolytic stability	[44]
*L*,*D‐TA* (**3**)	F*l*P*l*IGR*v*L*s*G*i*L	Reduced activity versus MRSA and VRE; alteration of the helical turn	[44]
*TB_kkG6k* (**4**)	kkLLPIVkNLLkSLL	No significant change in activity upon stereoinversion at these positions	[77]
*RI‐TL* (**5**)	*lirGlfksfwqvf*	Reduced activity; disrupted amphipathic organization	[70]
*D‐TL* (**6**)	*fvqwfskflgril*	Comparable activity to TL; global stereoinversion tolerated	[70]
[*Pro* ^3^,*D‐Leu* ^9^]*TL* (**7**)	FV**P**WFSKF*l*GRIL	Comparable antimicrobial activity; markedly reduced hemolysis	[41]
[*Pro* ^3^,*D‐Lys* ^7^]*TL* (**8**)	FV**P**WFS*k*FLGRIL	Reduced antimicrobial activity	[41]
[*Pro* ^3^,*D‐Phe* ^8^]*TL* (**9**)	FV**P**WFSK*f*LGRIL	Reduced antimicrobial activity	[41]
[*Pro* ^3^,*D‐Arg* ^11^]*TL* (**10**)	FV**P**WFSKFLG*r*IL	Reduced antimicrobial activity	[41]
[*Pro* ^3^,*D‐Ile* ^12^]*TL* (**11**)	FV**P**WFSKFLGR*i*L	Reduced antimicrobial activity	[41]
[*Pro* ^3^,*D‐Leu* ^13^]*TL* (**12**)	FV**P**WFSKFLGRI*l*	Reduced antimicrobial activity	[41]
[*Pro* ^3^,*D‐Nle* ^10^]*TL* (**13**)	FV**P**WFSKF*l*‐*nle*‐RIL	Minimal impact on activity	[41]
[*Pro* ^3^,*D‐Lys* ^10^]*TL* (**14**)	FV**P**WFSKF*lk*RIL	Increased antimicrobial activity	[41]
[*Pro* ^3^,*D‐Leu* ^9^,*D‐Trp* ^10^]*TL* (**15**)	FV**P**WFSKF*lw*RIL	Increased antimicrobial activity	[41]
*Ta A1* (**16**)	**A**LPLIGRVLSGIL	Reduced activity (MIC versus *S. aureus* 40 µM); increased hemolysis; increased helicity	[78]
*Ta A5* (**17**)	FLPL**A**GRVLSGIL	Reduced activity (MIC versus *S. aureus* 40 µM); low hemolysis (<0.5%)	[78]
*Ta A12* (**18**)	FLPLIGRVLSG**A**L	Reduced activity (MIC versus *S. aureus* 40 µM); low hemolysis (<0.5%)	[78]
*A5*,*12 TA* (**19**)	FLPL**A**GRVLSG**A**L	Inactive; increased hemolysis	[78]
*Ta A7* (**20**)	FLPLIG**A**VLSGIL	Increased antimicrobial activity *versus S. aureus*; increased helicity	[78]
*Ta A3* (**21**)	FL**A**LIGRVLSGIL	Increased antimicrobial activity *versus S. aureus*; increased helicity	[78]
*Ta A10* (**22**)	FLPLIGRVL**A**GIL	Increased antimicrobial activity *versus S. aureus*; increased hemolysis; increased helicity	[78]
*Ta A11* (**23**)	FLPLIGRVLS**A**IL	Increased antimicrobial activity *versus S. aureus*; increased hemolysis; increased helicity	[78]
*K1TA* (**24**)	**K**LPLIGRVLSGIL	Inactive *versus S. aureus*; amphipathicity disrupted	[53]
*K1‐D‐TA* (**25**)	***k**lpligrvlsgil*	Inactive *versus S. aureus*	[53]
*K7TA* (**26**)	FLPLIG**K**VLSGIL	Reduced activity *versus S. aureus*	[53]
*DTCit* (**27**)	FLPLIG‐**Cit**‐VLSGIL	Inactive *versus A. xylosoxidans*	[79]
*DTOrn* (**28**)	FLPLIG‐**Orn**‐VLSGIL	Reduced activity *versus S. aureus*	[79]
*DTDab* (**29**)	FLPLIG‐**Dab**‐VLSGIL	Reduced activity *versus S. aureus*	[79]
*DTDap* (**30**)	FLPLIG‐**Dap**‐VLSGIL	Reduced activity *versus S. aureus*; reduced cytotoxicity on fibroblastic cells	[79]
*E7TA* (**31**)	FLPLIG**E**VLSGIL	Inactive *versus S. aureus*	[44]
*AcTA* (**32**)	**Ac**‐FLPLIGRVLSGIL	Increased activity *versus S. aureus*; increased helicity	[44]
*TAc* (**33**)	FLPLIGRVLSGIL‐**OH**	Inactive *versus S. aureus*	[80]
*TAd* (**34**)	[**2×**(FLPLIGRVLSGILAGG)]‐**DAPPA‐G‐COOH**	Increased activity *versus S. aureus*; Increased hemolysis	[80]
*TB_P3A* (**35**)	LL**A**IVGNLLKSLL	Increased activity *versus S. aureus and S. epidermidis* (MIC 10–20 µg/mL)	[81]
*TB_N7A* (**36**)	LLPIVG**A**LLKSLL	Increased activity *versus S. aureus and S. epidermidis* (MIC 10–20 µg/mL)	[81]
*TB_S11A* (**37**)	LLPIVGNLLK**A**LL	Increased activity *versus S. aureus and S. epidermidis* (MIC 10–20 µg/mL)	[81]
*TB_G6A* (**38**)	LLPIV**A**NLLKSLL	Increased activity *versus S. aureus and S. epidermidis* (MIC of 5 and 10 µg/mL)	[81]
*TB_G6AS11A* (**39**)	LLPIV**A**NLLK**A**LL	Not improvement in antimicrobial activity	[81]
*TB_YK* (**40**)	**KKY**LLPIVGNLLKSLL	Reduced antimicrobial activity	[82]
*TB_KKG6A* (**41**)	**KK**LLPIV**A**NLLKSLL	Increased activity *versus E. coli*; reduced hemolysis	[83]
*TB_KKG6K* (**42**)	**KK**LLPIV**K**NLLKSLL	Increased activity *versus E. coli* (MIC 0.2 µM) and *S. epidermidis* (MIC 0.025 µM)	[83]
*P3K‐TF* (**43**)	FL**K**LIGKVLSGIL	Increased activity *versus E. coli* (MIC 4 µg/mL) and *S. epidermidis* (MIC 2 µg/mL)	[69]
*G6K‐TF* (**44**)	FLPLI**K**KVLSGIL	Increased activity	[69]
*S10K‐TF* (**45**)	FLPLIGKVL**K**GIL	Increased activity; increased hemolysis	[69]
*G11K‐TF* (**46**)	FLPLIGKVLS**K**IL	Not improved activity; increased hemolysis	[69]
*P3KG11K‐TF* (**47**)	FL**K**LIGKVLS**K**IL	Not improved in activity; Increased hemolysis	[69]
*F5A‐TL* (**48**)	FVQW**A**SKFLGRIL	Reduced hydrophobicity; reduced antimicrobial activity	[84]
*F58‐TL* (**49**)	FVQWFSK**A**LGRIL	Reduced antimicrobial activity; decreased cytotoxicity	[84]
*F5*,*8A‐TL* (**50**)	FVQW**A**SK**A**LGRIL	Reduced antimicrobial activity; reduced anti‐endotoxin activity	[84]
*Peptide 7* **(51**)	FV**(** ***cis*‐4NH_2_‐Pro** **)**WFSKFLGRIL	Not improved activity; increased hemolysis	[85]
*Arg* ^3^ *‐TL* (**52**)	FV**R**WFSKFLGRIL	Increased anti Gram‐negative activity; reduced hemolysis	[70]
*Arg* ^7^ *‐TL* (**53**)	FVQWFS**R**FLGRIL	Reduced hemolysis	[70]
*Arg* ^3^,*Arg* ^7^ *TL* (**54**)	FV**R**WFS**R**FLGRIL	Reduced antimicrobial activity	[70]
*Q3K‐TL* (**55**)	FV**K**WFSKFLGRIL	Increased antimicrobial activity; increased anti‐endotoxin activity	[70]
*Q3*,*F8K‐TL* (**56**)	FV**K**WFSK**K**LGRIL	Reduced antimicrobial activity; reduced cytotoxicity versus human cells	[86]
*SW*,*Q3*,*F8K‐TL* (**57**)	FV**KS**F**W**K**K**LGRIL	Increased anti Gram‐negative activity	[86]
*Lys* ^11^ *‐TL* (**58**)	FVQWFSKFLG**K**IL	Reduced hemolysis	[70]
*Orn* ^11^ *‐TL* (**59**)	FVQWFSKFLG‐**Orn**‐IL	Reduced antimicrobial; reduced hemolysis	[70]
*Pro* ^3^,*Orn* ^11^ *‐TL* (**60**)	FV**P**WFSKFLG‐**Orn**‐IL	Improved antimicrobial activity	[70]
*Pro* ^3^,*Lys* ^10^ *‐TL* (**61**)	FV**P**WFSKF*l* **K**RIL	Increased antimicrobial activity; reduced cytotoxicity versus human cells and erythrocytes	[87]
*TL‐1* (**62**)	FVQW**W**SK**W**LGRIL	Increased hydrophobicity	[88]
*TL‐2* (**63**)	FV**R**W**W**SK**W**L**R**RIL	Increased antimicrobial activity; increased anti‐inflammatory activity	[88]
*TL‐3* (**64**)	FV**R**W**W**S**RW**L**R**RIL	Not improved activity	[88]
*TL‐4* (**65**)	FV**K**W**W**S**RW**L**KK**IL	Reduced antimicrobial activity; reduced toxicity	[88]
*Hydrophobicity‐driven modifications:*			
*W1‐TA* (**66**)	**W**LPLIGRVLSGIL	Increased antimicrobial activity (MIC value versus *MRSA* = 0.5 µM);	[44]
*DT4F* (**67**)	**Phe** **(** **4F** **)**LPLIGRVLSGIL	Increased anti Gram‐negative activity (MIC value *versus P. aeruginosa* = 80 µg/mL)	[89]
*L5*,*12‐TA* (**68**)	FLPL**L**GRVLSG**L**L	Increased antimicrobial activity	[53]
*TAD‐5*,*12L* (**69**)	*flpl**l**grvlsg**l**l*	Increased antimicrobial activity; Increased hemolysis	[90]
*TB_G6I* (**70**)	LLPIV**I**NLLKSLL	Increased anti Gram‐positive activity	[81]
*TB_G6V* (**71**)	LLPIV**V**NLLKSLL	Not improved activity	[81]
*TB_L1FK* (**72**)	**F**LPIVG**L**LKSLL**K**	Expanded antimicrobial spectrum	[91]
*TB_KKP3K*(*Pal*)*G6A* (**73**)	**KK**LL**K** **(** **Pal** **)**IV**A**NLLKSLL	Reduced antimicrobial activity	[83]
*TB_KKP3K*(*Oct*)*G6A* (**74**)	**KK**LL**K** **(** **Oct** **)**IV**A**NLLKSLL	Reduced antimicrobial activity; promoted self‐aggregation	[83]
*TB_KKG6AN7K*(*Pal*) (**75**)	**KK**LLPIV**AK** **(** **Pal** **)**LLKSLL	Reduced antimicrobial activity; promoted self‐aggregation	[83]
*TB_KKG6AN7K*(*Oct*) (**76**)	**KK**LLPIV**AK** **(** **Oct** **)**LLKSLL	Reduced antimicrobial activity	[83]
*TB_KKG6K*(*Ac*) (**77**)	**KK**LLPIV**K(Ac)**NLLKSLL	Increased antimicrobial activity	[83]
*TB_KKG6K*(*DAVA*) (**78**)	**KK**LLPIV**K(DAVA)**NLLKSLL	Increased antimicrobial activity	[83]
*TB_KKG6K*(*Ahx*) (**79**)	**KK**LLPIV**K(Ahx)**NLLKSLL	Increased antimicrobial activity	[83]
*TB_KKG6K*(*BrHx*) (**80**)	**KK**LLPIV**K(BrHx)**NLLKSLL	Increased antimicrobial activity	[83]
*TB_KKG6K*(*Aund*) (**81**)	**KK**LLPIV**K(Aund)**NLLKSLL	Increased antimicrobial activity	[83]
[*Nle* ^1^,*Pro* ^3^,*D‐Leu* ^9^,*D‐Lys* ^10^]*TL* (**82**)	**Nle**‐V**P**WFSKF*lk*RIL	Increased anti Gram‐negative activity	[92]
*TLP‐1* (**83**)	F**L**QWFSKFLGRIL	Increased SARS‐CoV‐2 Mpro inhibition (IC_50_ = 11 µM)	[93]
*TLP‐2* (**84**)	**SAYW**QWFSKFLGRIL	Increased SARS‐CoV‐2 Mpro inhibition (IC_50_ = 7 µM)	[93]
*TLP‐3* (**85**)	**SAFW**QWFSKFLGR	Increased SARS‐CoV‐2 Mpro inhibition (IC_50_ = 7 µM)	[93]
*RB‐142* (**86**)	F**F**PWFSKFLGRIL	Enhanced antiviral activity	[94]
*RB‐143* (**87**)	F**(4‐Cl)F**PWFSKFLGRIL	Enhanced antiviral activity	[94]
*RB‐144* (**88**)	F**(3,4** **‐** **Cl_2_)F**PWFSKFLGRIL	Enhanced antiviral activity	[94]
*RB‐145* (**89**)	F‐**Nal(2)**‐PWFSKFLGRIL	Enhanced antiviral activity	[94]
*RB‐146* (**90**)	F‐**Nal(1)**‐WFSKFLGRIL	Enhanced antiviral activity	[94]
[*Pro* ^3^]*TL* (**91**)	FV**P**WFSKFLGRIL	Not improved in antimicrobial activity; disrupted helical structure	[70]
[*Leu* ^2^,*Pro* ^3^]*TL* (**92**)	F**LP**WFSKFLGRIL	Reduced antimicrobial activity	[70]
[*Pro* ^3^,*Pro* ^10^]*TL* (**93**)	FV**P**WFSKFL**P**RIL	Markedly reduced antimicrobial activity; disrupted hydrophobic surface	[87]
[*Pro* ^3^,*D‐Pro* ^10^]*TL* (**94**)	FV**P**WFSKFL*p*RIL	Markedly reduced antimicrobial activity	[87]
*Compound 3* (**95**)	FV**(** ***cis*** **‐** **4F)**PWFSKFLGRIL	Reduced antimicrobial activity (2‐fold increase of MIC values)	[85]
*Compound 4* (**96**)	FV**(** **4,4‐** **F_2_)** **P**WFSKFLGRIL	Reduced antimicrobial activity	[85]
*Compound 5* (**97**)	FV**(** ***cis*** **‐** **4CF_3_** **)P**WFSKFLGRIL	Reduced antimicrobial activity; Increased self‐aggregation	[85]
*Compound 6* (**98**)	FV**(** ***cis*‐** **4MeS)P**WFSKFLGRIL	Reduced anti Gram‐negative activity; increased self‐aggregation	[85]
*Compound 8* (**99**)	FV**(** ***trans‐*** **4Chx)P**WFSKFLGRIL	Reduced antimicrobial activity	[85]
*Compound 9* (**100**)	FV**(** ***trans‐*** **4Ph)P**WFSKFLGRIL	Reduced antimicrobial activity; increased self‐aggregation	[85]
*Compound 10* (**101**)	FV**(** ***trans‐*** **4Bn)P**WFSKFLGRIL	Reduced anti Gram‐negative activity	[85]
*Compound 11* (**102**)	FV**(** ***cis‐*** **4PhO)P**WFSKFLGRIL	Reduced anti Gram‐negative activity	[85]
*F5L‐TL* (**103**)	FVQW**L**SKFLGRIL	Increased anti‐endotoxic activity; reduced hemolysis	[84]
*F8L‐TL* (**104**)	FVQWFSK**L**LGRIL	Increased anti‐endotoxic activity; reduced hemolysis	[84]
*F5*,*8L‐TL* (**105**)	FVQW**L**SK**L**LGRIL	Increased anti‐endotoxic activity; increased hemolysis	[84]
[*Leu* ^10^]*TL* (**106**)	FVQWFSKFL**L**RIL	Reduced antimicrobial activity (MIC > 48 µM); increased hemolysis (78% at 3 µM)	[70]
*β‐leucine* (**107**)	FVQWFSKF ***β*** **L**RIL	Not improved in activity; loss of helical content	[95]
*TL‐1* (**108**)	**LL**QW**L**SK**L**LGR**L**L	Reduced antimicrobial activity	[70]
*TL*‐*2* (**109**)	**LL**QW**L**SK**L**LGR**W**L	Reduced antimicrobial activity	[70]
[*Pro* ^3^,*Nle* ^10^]*TL* (**110**)	FV**P**WFSKF*l*‐**Nle**‐RIL	Increased antimicrobial activity (MIC versus *S. aureus* 1.56–3.12 µM)	[87]
[*Pro* ^3^,*Trp* ^10^]*TL* (**111**)	FV**P**WFSKF*l* **W**RIL	Increased antimicrobial activity (MIC versus *A. baumannii* 3.12–6.25 µM)	[87]
[*Pro* ^3^,*Aic* ^10^]*TL* (**112**)	FV**P**WFSKF*l*‐**Aic**‐RIL	Increased anti Gram‐positive activity	[87]
*Noncanonical amino acid substitutions and other modifications:*			
*DTTyr1* (**125**)	**Y**LPLIGRVLSGIL	Reduced antimicrobial activity	[89]
*G3TA* (**126**)	FL**G**LIGRVLSGIL	Not improved activity; increased hemolysis	[44]
*Q3TA* (**127**)	FL**Q**LIGRVLSGIL	Not improved activity; increased hemolysis	[96]
*DTThr10* (**128**)	FLPLIGRVL**T**GIL	Not improved anti Gram‐positive activity; increased anti Gram‐negative activity	[89]
*DTTyr10* (**129**)	FLPLIGRVL**Y**GIL	Reduced antimicrobial activity	[89]
(*6–1*)(*7–13*)*TA* (**130**)	**GILPLF**RVLSGIL	Reduced antimicrobial activity	[97]
[*Pro* ^3^,*D-Hyp* ^10^]*TL* (**131**)	FV**P**WFSKF*l*‐**Hyp**‐RIL	Not improved activity	[87]
[*Pro* ^3^,*D‐Hyp* ^10^]*TL* (**132**)	FV**P**WFSKF*l*‐***hyp***‐RIL	Reduced anti Gram‐negative activity	[87]
*TempL SCR‐1* (**133**)	F**L**Q**FW**SK**GVF**RIL	Reduced antimicrobial activity; reduced helical content	[84]
*TempL SCR‐2* (**134**)	F**L**Q**LW**SK**GVL**RIL	Reduced antimicrobial activity	[84]
*Peptide 3* (**135**)	GFKS*l*WPRFVFLI	Loss of anti‐inflammatory properties	[98]
*Peptide 4* (**136**)	*k*FKS*l*WPRFVFLI	Loss of anti‐inflammatory properties	[98]

a Residues shown in bold indicate amino acid substitutions relative to the native sequence; residues in lowercase italics denote amino acids in the D‐configuration; underlined sequences indicate retro‐inverso analogs.

### 3.1 Backbone Modifications: Inverso/Retro‐Inverso Analogs and D‐Amino Acid Incorporation

Structural modifications aimed at improving the pharmacological profile of AMPs have also explored sequence inversion and backbone reorganization [53]. In parallel, the incorporation of D‐amino acids has been extensively employed to probe the stereochemical contribution to activity, leading to the development of several derivatives containing D‐residue substitutions.

In the case of TA, Wade et al. investigated both sequence inversion and global stereochemical inversion. The reverse‐sequence analog *Rev‐TA* (**1**) displayed a marked loss of antimicrobial activity against Gram‐positive strains compared with the native peptide, with MIC values against *MRSA* K35 increasing from 2.5 to >10 μM and against *VRE* 29212 from 5 to >10 μM [44]. This reduction has been attributed to perturbations of the helical dipole and to changes in the spatial distribution of residues induced by sequence inversion. By contrast, the all‐D analog *D‐TA* (**2**) retained antimicrobial activity comparable to that of TA, showing similar or even slightly improved potency against Gram‐positive strains such as *MRSA* 128 (MIC from 2.7 to 1.3 μM) and *E. faecium* CCUG (from 21.5 to 6.9 μM) [76]. In addition, compound **2** exhibited bactericidal activity against *Propionibacterium acnes* (MIC 2.4–3.6 μM) together with enhanced resistance to proteolytic degradation [44]. Altogether, these findings indicate that TA activity is largely nonchiral and primarily governed by membrane‐disruptive interactions rather than stereospecific recognition. Consistent with this interpretation, the introduction of an alternating L,D stereochemical pattern in the analog *L,D‐TA* (**3**) resulted in a significant reduction in activity, with MIC values generally 2‐ to 4‐fold higher than those of the native peptide against both Gram‐positive and Gram‐negative strains (e.g., *MRSA* K35 from 2.5 to >10 μM, *VRE* 29212 from 5 to >10 μM) [44]. This loss of activity has been linked to alterations in the helical turn, suggesting that disruption of the native stereochemical pattern compromises peptide conformation and, consequently, biological function [44].

A similar approach was applied to TB, where the role of stereochemistry was explored by introducing additional D‐amino acids at selected positions. Specifically, guided by previous SAR studies, two D‐Lys residues were inserted at the *N*‐terminus and one D‐Lys replaced Gly^6^, yielding the analog *TB_kkG6k* (**4**), with the aim of increasing resistance to proteolytic degradation, exhibit increased antimicrobial efficacy, and reducing hemolytic activity [62, 77]. Notably, this analog retained significant antimicrobial and antifungal activity (MIC 14.4 μM against *S. aureus* and 1.8 μM against *C. albicans*), indicating that the introduction of stereoinverted lysine residues was not a critical determinant of the TB mechanism of action [77].

The impact of backbone inversion and stereochemical modifications has been even more extensively investigated for TL. Mangoni et al. first reported both a retro‐inverso analog (*RI‐TL*, **5**) and an all‐D analog (*D‐TL*, **6**) [70]. While compound **5** showed reduced antimicrobial activity, evidenced by increased MIC values against *Staphylococcus epidermidis* (from 3 to 6 μM) and *P. aeruginosa* (from 24 to >48 μM), analog **6** largely preserved the activity of the native peptide (e.g., MIC 6 μM against *A. baumannii*) [70]. These results mirror those observed for TA, reinforcing the notion that stereochemical inversion of the entire sequence is compatible with antimicrobial function in membrane‐active peptides. Building on these findings, Grieco et al. investigated the role of local stereochemistry through a targeted D‐scan on a TL analog containing a Gln to Pro substitution at position 3, which promotes an α‐helical conformation spanning residues 6–13 [70, 78]. Within this helical region (Lys^7^‐Leu^13^), individual residues were systematically replaced with their D‐enantiomers, acting as helix‐disrupting elements and enabling identification of positions where stereochemical inversion modulates activity and toxicity. Among these, position 9 emerged as particularly significant. The analog [*Pro* ^3^,*D‐Leu* ^9^]*TL* (**7**) retained antimicrobial activity comparable to native TL (MIC 3 μM against *C. albicans* and 12 μM against *E. coli*), while exhibiting a dramatic reduction in hemolytic activity (7% at 12 μM) [78]. This identifies Leu^9^ as a key stereochemical hotspot for improving selectivity without compromising potency. NMR studies further confirmed preservation of the α‐helical conformation (residues 6–13) in SDS and DPC micelles, indicating that stereoinversion at this position is compatible with maintenance of the bioactive fold while reducing peptide toxicity. In contrast, inversion at other positions, such as Lys^7^ (**8**), Phe^8^ (**9**), Arg^11^ (**10**), Ile^12^ (**11**) and Leu^13^ (**12**), generally led to reduced antimicrobial activity (2‐ to 8‐fold higher MIC values) [41, 78]. Further insights into positional tolerance were provided by Merlino et al., who focused on residue 10 by replacing Gly with different amino acids in both L and D configurations [87]. Substitutions at this position generally yielded analogs with comparable antimicrobial activity, indicating that it is relatively permissive to variations in both side chain and stereochemistry. A similar trend was observed for antifungal activity, where substitutions with hydrophobic, polar, and aromatic residues, such as D‐Nle (**13**), D‐Lys (**14**), and D‐Trp (**15**), showed minimal differences between L and D variants. In particular, peptide **14** also showed promising activity against a panel of clinically relevant Gram‐negative strains and yeast [87]. These results highlight position 10 as a stereochemically tolerant site, in contrast to more sensitive residues within the helical region, such as Leu^9^, which can be strategically targeted to enhance selectivity while preserving antimicrobial potency.

### 3.2 Side Chain Modifications: Alanine Scanning and Cationic Residues/Net Charge Modulation

Alanine‐scanning studies have been extensively employed to elucidate SAR in temporins, allowing the identification of key residues as well as regions that are more tolerant to substitution. In parallel, systematic modulation of the number, nature, and spatial distribution of cationic residues has been widely explored. Together, these approaches highlight how both side‐chain identity and overall net charge represent central determinants in tuning antimicrobial activity and selectivity in temporin analogs.

In the case of TA, alanine‐scanning studies first delineated the presence of a conserved hydrophobic core that is essential for activity. Substitution of selected residues, including Phe^1^ (i.e., *Ta A1*, compound **16**), Ile^5^ (i.e., *Ta A5*, compound **17**), and Ile^12^ (i.e., *Ta A12*, compound **18**) consistently resulted in a marked reduction in antimicrobial activity, indicating that these positions are critical for maintaining the structural features required for bioactivity [70]. Notably, although some substitutions (e.g., peptide **16**) increased α‐helical content, particularly in DPC micelles, this structural stabilization did not translate into improved function but rather into reduced antimicrobial activity (e.g., MIC against *S. aureus* from 5 to >40 μM) and increased hemolysis. Similarly, peptides **17** and **18** displayed a marked loss of activity while maintaining negligible hemolytic effects, and the double mutant *A5*,*12TA* (compound **19**) was completely inactive, confirming that disruption of the hydrophobic core is highly detrimental [70, 78]. Beyond hydrophobic residues, alanine scanning also underscored the importance of specific polar and charged positions. In particular, substitution of Arg^7^ (i.e., *Ta A7*, compound **20**) caused a dramatic loss of antimicrobial activity (MIC > 40 μM) accompanied by a substantial increase in hemolysis (up to 28% at 5 μM), highlighting its key role in balancing activity and selectivity. In contrast, residues such as Pro^3^ (i.e., *Ta A3*, compound **21**), Gly^6^, Ser^10^ (*Ta A10*, compound **22**) and Gly^11^ (i.e., *Ta A11*, compound **23**) were more permissive to substitution [70]. Removal of the helix‐breaking Pro^3^ increased α‐helicity and improved activity against Gram‐positive strains, while substitutions at positions 10 and 11 also enhanced antimicrobial potency. However, these gains were accompanied by increased hemolytic activity, suggesting that enhanced helicity may come at the expense of selectivity [70]. These observations naturally led to a second level of optimization focused on modulating the net positive charge. Interestingly, an increase in charge did not universally improve the activity. For example, substitution of Phe^1^ with Lys (K1TA, **24**) and its all D‐analog (*k1D‐TA*, compound **25**) resulted in complete loss of activity against selected clinically relevant Gram‐positive and Gram‐negative bacteria, likely due to disruption of the amphipathic helical arrangement [53]. This highlights that preservation of the hydrophobic‐hydrophilic balance is as critical as charge itself. A particularly instructive case is Arg^7^, whose role was further dissected through systematic substitutions. Replacement with Lys (i.e., K7TA, compound **26**) led to reduced antibacterial activity, likely due to the weaker electrostatic interactions of the lysine ε‐amino group compared to the guanidinium group of arginine [53]. This interpretation was reinforced by substitutions with residues differing in charge and side‐chain length: neutral citrulline (compound **27**) abolished activity, while shorter cationic analogs such as ornithine (compound **28**), diaminobutyric acid (compound **29**), and diaminopropionic acid (compound **30**) markedly reduced potency [79]. Interestingly, shortening the side chain (as in peptide **30**) also reduced hemolysis, suggesting a possible route to improved selectivity. Consistently, introduction of a negatively charged residue (i.e., *E7TA*, compound **31**) resulted in complete loss of activity [44]. Collectively, modulation of net charge further confirmed this delicate balance. *N*‐terminal acetylation (i.e., *AcTA*, compound **32**), which reduces effective charge, surprisingly improved activity against *E. faecalis*, whereas *C*‐terminal deamidation (i.e., *TAc*, compound **33**), lowering the net charge from +2 to +1, led to complete loss of activity. Conversely, increasing charge through dimerization (i.e., *TAd*, compound **34**), with +4 overall net charge, enhanced antibacterial potency but also increased hemolysis [53, 80].

A comparable SAR framework has been applied to TB, where alanine scanning revealed a more localized tolerance to substitution. Residues such as Pro^3^, Asn^7^, and Ser^11^ could be replaced with minimal or even beneficial effects on activity (analogs **35–37**), whereas substitution of Gly^6^ (compound **38**) significantly enhanced antimicrobial potency [81]. Interestingly, the double mutant *TB_G6A_S11A* (compound **39**) did not provide further improvement, indicating that position 6 is the primary contributor to this effect. In contrast, substitutions of hydrophobic residues across the sequence and of Lys^10^ resulted in reduced activity, confirming the importance of both the hydrophobic face and key cationic residues for membrane interaction [81]. Building on these findings, modulation of net charge proved particularly effective for TB. Introduction of additional residues at the *N*‐terminus, as in *TB‐YK* (compound **40**), enhanced activity, especially against Gram‐negative bacteria, and conferred synergistic and anti‐inflammatory properties [82]. A more pronounced improvement was achieved with *TB_KKG6A* (compound **41**), where increased charge combined with the G6A substitution resulted in a dramatic expansion of the antimicrobial spectrum and potency, along with reduced hemolysis and additional antibiofilm and immunomodulatory properties [99]. Further optimization identified position 6 as highly permissive, as substitution with Lys led to a highly potent analog (*TB_KKG6K*, compound **42**), underscoring how the combined tuning of charge and residue positioning can markedly enhance activity [81].

These principles were further validated in TF, where increasing net positive charge through lysine substitutions produced position‐dependent effects [69]. Several peptides were developed through substitution at Pro^3^ (compound **43**), Gly^6^ (compound **44**), Ser^10^ (compound **45**), Gly^11^ (compound **46**), and by double substitution at positions 3 and 11 (compound **47**). Among the analogs, *G6K‐TF* (compound **44**) displayed the most significant improvement in activity while remaining nonhemolytic, whereas modifications at other positions led to more modest gains or increased cytotoxicity. Notably, increased helicity did not directly correlate with activity, indicating that an optimal balance between charge distribution, amphipathicity, and conformational flexibility is required for effective membrane interaction.

Extensive SAR studies on TL have provided a more complex and nuanced picture. Alanine substitutions targeting the phenylalanine zipper motif (positions 5 and 8) demonstrated its crucial role in maintaining antimicrobial activity. In particular, the substitution of Phe^5^ (*F5A‐TL*, compound **48**), Phe^8^ (*F8A‐TL*, compound **49**), as well as the double mutant (*F5*,*8A‐TL*, compound **50**), led to disruption of this hydrophobic motif led to reduced potency (e.g., against *P. aeruginosa*, MIC value from 15 to >60 µM, while against *S. aureus* and *Bacillus subtilis* MIC value from 15 to 30 µM), but also decreased cytotoxicity (%viability of macrophage cells >80% at 10 μM), highlighting a trade‐off between activity and safety [84]. Beyond hydrophobic determinants, modulation of cationic residues and net charge has been widely explored in TL. In some cases, introducing positively charged residues improved activity and selectivity, as observed for the (*cis*‐4‐amino)Pro substitution at position 3 (compound **51**) [85]. However, simply increasing net charge is not always beneficial. Replacement of Gln^3^ with Arg (compound **52**) or combined substitutions (compound **54**) failed to improve activity (e.g., compound **53** showed an MIC value of 48 µM against *E. coli* D21) and often increased toxicity, underscoring the importance of charge distribution rather than absolute charge [44]. More refined strategies have demonstrated that specific positions can be exploited to optimize this balance. For example, substitution of Gln^3^ with Lys (compound **55**) improved antimicrobial and anti‐endotoxic activity while reducing hemolysis, whereas further charge increase (compound **56**) reduced cytotoxicity but at the expense of potency. Interestingly, reorganization of residue distribution, as in the *SW*,*Q3K*,*F8K‐TL* analog (compound **57**), restored and even enhanced activity [86, 100]. Additional studies further emphasized the role of individual cationic residues. Modifications at position 11 showed that replacing Arg with Lys (compound **58**) or Orn reduced both activity and toxicity, confirming the importance of the guanidinium group for strong membrane (compound **59**) interactions [70]. However, in different structural contexts, such as proline‐containing analogs in which the substitution with Orn at position 11 (compound **60**) lead to improved activity. Similarly, the introduction of Lys at position 10 in the proline‐containing analog (compound **61**) also enhanced activity against selected clinically relevant Gram‐positive and Gram‐negative strains [87], illustrating the context‐dependent nature of these effects. Finally, more extensive charge modulation strategies confirmed that increasing net positive charge can enhance activity but often at the cost of selectivity [88]. For instance, starting from the Trp‐enriched analog *TL‐1* (compound **62**), the introduction of additional cationic residues afforded *TL‐2* (compound **63**), which displayed improved antimicrobial and anti‐inflammatory properties, whereas further charge increase (i.e., *TL‐3*, compound **64**) did not yield additional benefits, and substitution of Arg with Lys (i.e., *TL‐4*, compound **65**) reduced both activity and toxicity.

### 3.3 Hydrophobicity‐Driven Modifications

Hydrophobicity represents one of the major physicochemical determinants governing temporin activity, as it directly influences peptide folding, membrane insertion, aggregation propensity, antimicrobial potency, and cytotoxicity. Accordingly, several studies have explored hydrophobic modifications in temporin analogs, with the aim of defining how changes in hydrophobic residue identity, side‐chain bulk, aromaticity, and lipidation affect biological activity.

In TA, hydrophobic modulation has been mainly investigated at the *N*‐terminus, where Phe^1^ plays a key role in preserving the amphipathic character of the peptide. Replacement of Phe^1^ with Trp, yielding *W1‐TA* (compound **66**), maintained the aromatic and hydrophobic nature of this position and enhanced antimicrobial activity against selected strains, including *MRSA* (MIC from 2.7 to 0.6 μM) and *P. acnes* (MIC from 15 to 5 μM) [44]. A related strategy was pursued by Dimitrova et al., who introduced the nonproteinogenic fluorinated residue 4‐fluorophenylalanine at position 1, generating *DT4F* (compound **67**) [89]. This modification improved antimicrobial activity, particularly against Gram‐positive bacteria such as *B. subtilis* and *Alcaligenes oxydans*, and also enhanced activity against Gram‐negative strains such as *P. aeruginosa*, with up to a 4‐fold reduction in MIC values. In addition, peptide **67** displayed antiproliferative activity against cancer cell lines, including MCF‐7 and MDA‐MB‐231. However, the increased potency was accompanied by increased cytotoxicity on fibroblast cells and phototoxicity, highlighting once again the delicate balance between hydrophobic enhancement and safety. Hydrophobic tuning has also been explored at internal positions of TA. Replacement of Ile residues at positions 5 and 12 with Leu, yielding *L5*,*12‐TA* (compound **68**), enhanced antimicrobial activity against both Gram‐positive and Gram‐negative bacteria, including *S. aureus* and *E. faecium* (e.g., MIC value of 5.6 μM) [53]. Although Ile and Leu share similar physicochemical properties, these results indicate that subtle differences in side‐chain topology are sufficient to modulate peptide function. The same strategy applied to the all‐D analog, *TAD‐5*,*12L* (compound **69**), further improved antibacterial activity, completely inhibiting *S. aureus* at 2 μM [90]. However, this gain in potency was accompanied by increased cytotoxicity on keratinocyte cells, as reflected by reduced keratinocyte viability, again suggesting that strengthening hydrophobic interactions may improve activity at the expense of selectivity.

Hydrophobic modifications have also been extensively investigated in TB. Initial studies by Avitabile et al. focused on position 6, where the introduction of hydrophobic residues such as Ile and Val generated *TB_G6I* (compound **70**) and *TB_G6V* (compound **71**) [81]. These substitutions produced a moderate improvement in activity against Gram‐positive strains, with MIC values decreasing from 25 μg/mL for native TB to 10–20 μg/mL. However, comparison with the alanine‐substituted analog *TB_G6A* (compound **38**) showed that further increasing hydrophobicity at this position did not necessarily improve activity, since peptides **70** and **71** were less effective against *S. epidermidis*. Thus, position 6 appears to benefit from a limited increase in hydrophobic character rather than from maximal hydrophobic reinforcement. A more integrated modulation of hydrophobicity and charge was achieved in *TB_L1FK* (compound **72**), in which Leu^1^ was replaced with Phe, Asn^7^ with Leu, and an additional Lys was introduced at the *C*‐terminus [91]. This combination enhances membrane interactions and broadens the antimicrobial spectrum, particularly against Gram‐negative bacteria. Peptide **72** showed markedly improved activity against *P. aeruginosa* and Gram‐positive strains, together with rapid bactericidal kinetics and antibiofilm activity, while maintaining low cytotoxicity toward mammalian cells and hemolysis at active concentrations. This analog illustrates how hydrophobicity can be profitably increased when accompanied by appropriate charge compensation. Starting from the optimized *TB_KKG6A* analog (compound **41**), site‐specific acylation further clarified the position‐dependent impact of hydrophobic modifications [83]. Since residues 3, 6, and 7 are involved in membrane insertion, these positions were functionalized with hydrophobic chains of different lengths (compounds **73–81**). Acylation at positions 3 and 7 with long chains, such as palmitoyl or octanoyl groups, caused a marked loss of activity, indicating that excessive local hydrophobicity in these regions is detrimental. In contrast, modification at position 6 was more favorable. Analogs bearing short acyl chains at this site showed improved activity against *S. epidermidis* and *P. aeruginosa*. However, increasing chain length progressively reduced activity, likely because long hydrophobic chains promote self‐association and reduce conformational flexibility. These results indicate that position 6 is a privileged site for controlled hydrophobic tuning in TB.

**FIGURE 4:**
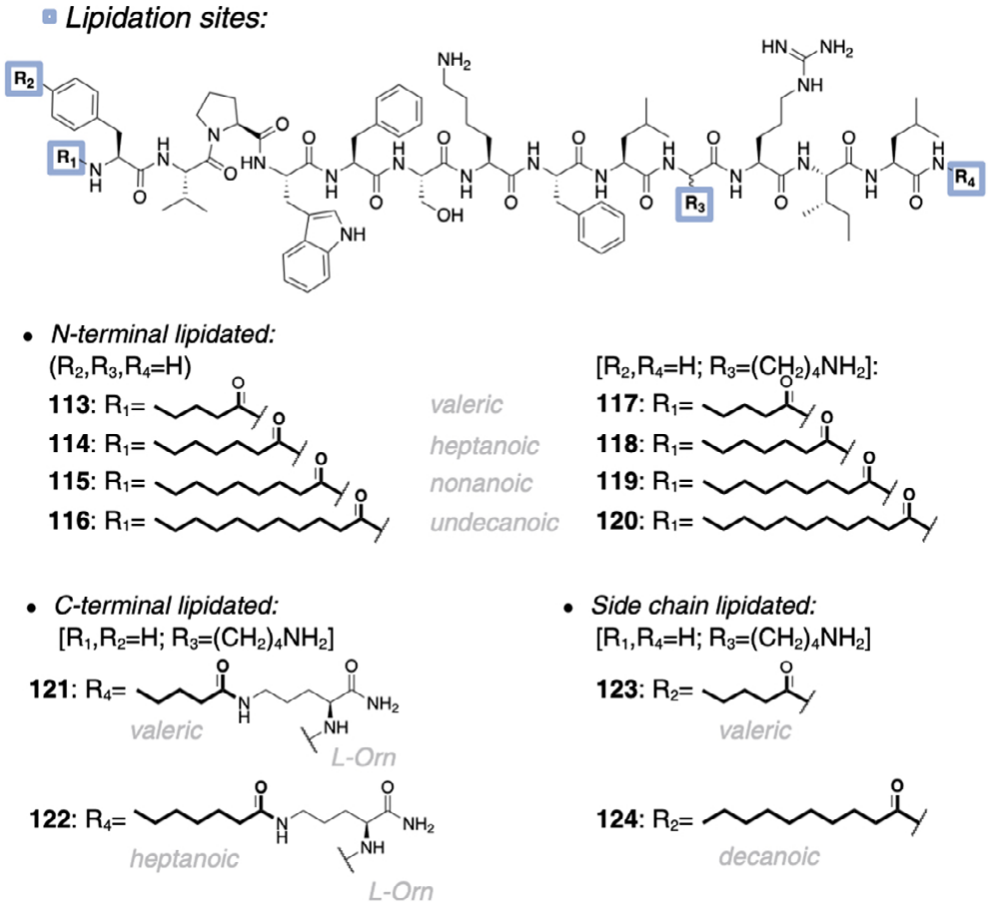
Chemical structures of lipidated temporin analogs bearing fatty acid conjugates at different positions of the peptide scaffold.

Hydrophobic modulation in TL has provided an even more complex picture, reflecting the strong contribution of hydrophobic clustering to its biological profile. One relevant determinant is the phenylalanine‐rich hydrophobic surface, often described as a phenylalanine zipper motif. Alanine substitutions at Phe^5^ and Phe^8^, yielding *F5A‐TL* (compound **48**), *F8A‐TL* (compound **49**), and *F5*,*8A‐TL* (compound **50**), reduced peptide hydrophobicity and caused a marked loss of antimicrobial activity [84]. At the same time, these analogs showed reduced anti‐endotoxin activity, hemolysis, and cytotoxicity toward mammalian cells. Thus, the phenylalanine zipper is essential for full TL potency but also contributes to its toxicity. Other position‐specific substitutions further confirmed that the effect of hydrophobicity depends strongly on its structural context. Replacement of Phe^1^ with Nle in a TL‐derived analog generated peptide **82**, which displayed increased hydrophobicity and enhanced activity against Gram‐negative strains (e.g., MIC value of 6.25 µM against *E. coli* and 3.12 µM against *A. baumannii*) [92]. Notably, antimicrobial activity was retained even when the peptide adopted a β‐aggregate secondary structure, indicating that TL activity is not strictly dependent on canonical α‐helical folding. At position 2, replacement of Val with Leu generated *TLP‐1* (compound **83**), which improved inhibitory activity against the SARS‐CoV‐2 main protease, with IC_50_ values decreasing from 39 to 11 μM [93]. Further redesign, including *N*‐terminal extension and incorporation of substrate‐mimicking motifs, afforded *TLP‐2* (compound **84**) and *TLP‐3* (compound **85**) with additional improvements in inhibitory activity (e.g., IC_50_ approximately 7 μM for compound **85**) [93]. Subsequent studies on proline‐containing TL analogs explored substitution of Val^2^ with aromatic residues, including Phe (compound **86**), 4Cl‐Phe (compound **87**), 3,4‐diCl‐Phe (compound **88**), Nal(2) (compound **89**), and Nal(1) (compound **90**), several of which showed antiviral activity against SARS‐CoV‐2, particularly analog **90** [94]. The introduction of Pro at position 3, yielding [*Pro* ^3^]*TL* (compound **91**), altered the spatial distribution of hydrophobic residues by disrupting the helical structure while maintaining activity and toxicity profiles comparable to native TL. Building on this scaffold, [*Leu* ^2^,*Pro* ^3^]*TL* (compound **92**) showed only a slight reduction in antimicrobial activity. In contrast, introduction of a second Pro residue at position 10, as in [*Pro* ^3^,*Pro* ^10^]*TL* (compound **93**) and [*Pro* ^3^,*D*‐*Pro* ^10^]*TL* (compound **94**), caused complete loss of activity, likely due to disruption of the hydrophobic surface required for efficient membrane interaction [70]. Position 3 was further explored using substituted proline residues bearing hydrophobic, aromatic, or fluorinated groups [85]. Fluorinated analogs such as *cis*‐4F‐Pro (compound **95**), 4,4‐diF‐Pro (compound **96**), and *cis*‐4CF_3_‐Pro (compound **97**) increased hydrophobicity but reduced antimicrobial activity. Similarly, aromatic and hydrophobic substituents, including *cis*‐4MeS‐Pro (compound **98**), *trans*‐4Chx‐Pro (compound **99**), *trans*‐4Ph‐Pro (compound **100**), *trans*‐4Bn‐Pro (compound **101**), and *cis*‐4PhO‐Pro (compound **102**), led to reduced potency, particularly against Gram‐negative bacteria. This loss of activity was attributed to increased peptide self‐aggregation, supporting the idea that excessive hydrophobicity may limit productive membrane interactions. The contribution of the phenylalanine zipper was further probed by replacing Phe^5^ and/or Phe^8^ with Leu, generating *F5L‐TL* (compound **103**), *F8L‐TL* (compound **104**), and *F5*,*8L‐TL* (compound **105**) [84]. These modifications increased hydrophobicity and improved anti‐endotoxic activity. The mono‐substituted analogs also showed moderately reduced hemolysis, suggesting improved selectivity, whereas the double mutant displayed increased hemolytic activity. Thus, partial hydrophobic remodeling can be beneficial, while excessive reinforcement of the hydrophobic face may impair membrane selectivity. Position 10 represents another sensitive site. Replacement of Gly^10^ with Leu, yielding [*Leu* ^10^]*TL* (compound **106**), caused a dramatic loss of antimicrobial activity and a marked increase in hemolysis [77]. This behavior was attributed to enhanced aggregation, indicating that excessive hydrophobicity at this position is particularly detrimental. Conversely, replacement of Leu^9^ and Gly^10^ with β‐leucine (compound **107**) preserved antimicrobial activity while abolishing α‐helical secondary structure, suggesting that alternative hydrophobic architectures may support activity even in the absence of classical helicity [95]. More extensive remodeling of the TL hydrophobic face also proved challenging. Replacement of Phe^1^, Val^2^, Phe^5^, Phe^8^, and Ile^12^ with Leu, generating *TL‐1* (compound **108**), reduced antimicrobial activity, although a modest improvement was observed against *P. aeruginosa*. Further replacement of Leu^12^ with Trp in *TL‐2* (compound **109**) caused additional loss of activity and increased toxicity, confirming that excessive hydrophobic bulk is generally unfavorable [70]. In contrast, more controlled hydrophobic modulation at position 10 within the optimized [*Pro* ^3^,*D‐Leu* ^9^]*TL* scaffold proved advantageous. Merlino et al. introduced hydrophobic residues such as Nle and Trp, yielding [*Pro* ^3^,*Nle* ^10^]*TL* (compound **110**) and [*Pro* ^3^,*Trp* ^10^]*TL* (compound **111**) [87]. These analogs showed improved antimicrobial activity compared with peptide **7**, particularly against *S. epidermidis* and *A. baumannii*. Moreover, peptide **111** showed promising activity against a selected panel of human clinical isolates of Gram‐positive bacteria, with MIC values of 6.25 μM. Similarly, incorporation of the constrained hydrophobic residue Aic at position 10, generating [*Pro* ^3^,*Aic* ^10^]*TL* (compound **112**), improved activity against Gram‐positive strains. These findings indicate that hydrophobicity at position 10 can be beneficial when introduced within an appropriate conformational and stereochemical context. Finally, lipidation strategies have been widely investigated in TL analogs as a means to increase hydrophobicity and modulate peptide localization, stability, and membrane affinity (Figure 4) [101]. In general, lipopeptides bearing long fatty acid chains, such as C17 and C20, were inactive, most likely because of excessive aggregation. In contrast, shorter chains, particularly C8‐C14, provided better antimicrobial activity against bacteria and *Candida* species, defining an optimal hydrophobicity window for lipidated TL derivatives. Importantly, chain length was not the only determinant: the site of lipidation was equally critical [102]. *N*‐ terminal lipidation of compound **7** generated compounds **113–116**, while lipidation of analog **14** afforded compounds **117–120**, both resulting in reduced antimicrobial activity, with MIC values against *S. aureus* increasing from 6.25 to 12.5–100 µM and against *Klebsiella pneumoniae* and *P. aeruginosa* from 50 to 100 µM [98, 102]. Conversely, modification at the para‐position of Phe^1^ preserved the overall cationicity while introducing a localized hydrophobic increase, resulting in improved biological profiles [102]. Conversely, modification at the *para*‐position of Phe^1^ preserved the overall cationicity while introducing a localized hydrophobic increase [98, 103]. In this context, *Pent1B* (compound **123**), bearing a short C5 lipid chain, showed improved antibacterial activity against *P. aeruginosa* (MIC value of 25 µM) and *K. pneumoniae* (MIC value of 6.25 µM) while retaining antifungal activity. Extension of the acyl chain, as in *Dec‐1B* (compound **124**), maintained a comparable antifungal profile [98, 103].

### 3.4 Noncanonical Amino Acid Substitutions and Other Modifications

Beyond classical substitutions and hydrophobic tuning, a variety of additional modifications, including sequence rearrangements and the introduction of noncanonical amino acids, have been explored to further elucidate the SAR of temporins.

In TA, several targeted substitutions have been performed to probe the contribution of specific side chains. For instance, replacement of Phe^1^ with Tyr (*DTTyr1*, compound **125**) resulted in a marked decrease in antimicrobial activity against both Gram‐positive and Gram‐negative bacteria [89]. Despite the structural similarity between these aromatic residues, the introduction of a polar hydroxyl group appears to perturb the delicate hydrophobic balance required for optimal activity. In contrast, position 3 was confirmed to be more permissive. Substitution of Pro^3^ with Gly (*G3TA*, compound **126**) or Gln (*Q3TA*, compound **127**) yielded analogs that retained antimicrobial activity, indicating that this position tolerates significant variation [44, 96]. These results also suggest that removal of the helix‐disrupting Pro residue does not necessarily impair function, further supporting the idea that TA activity is not strictly dependent on a rigid helical structure. Modifications at position 10 provided additional nuance. Substitution of Ser^10^ with Thr or Tyr (*DTThr10*, compound **128** and *DTTyr10*, compound **129**) generally reduced antimicrobial activity, although peptide **128** retained activity against several strains and even showed improved potency against *B. subtilis*. This indicates that conservative modifications at this position may be tolerated, but only within a limited structural window [89]. More extensive perturbations of the sequence have been explored through backbone rearrangement. The analog (6–1)(7–13)*TA* (compound **130**), generated by sequence reorganization, exhibited a moderate reduction in antimicrobial activity (approximately 2‐fold higher MIC values) [97]. Structural studies revealed a disordered conformation in aqueous solution and a partially helical segment in TFE (residues 7–11), with both termini adopting β‐turn motifs stabilized by hydrogen bonds.

For TL, noncanonical substitutions have been particularly informative in probing the relationship between local conformation and biological function. Position 10, already identified as a sensitive site, has been extensively modified using conformationally constrained residues [79]. Replacement of Gly^10^ with hydroxyproline (Hyp) in either l or d configuration generated analogs [*Pro* ^3^,*D‐Hyp* ^10^]*TL* (compound **131**) and [*Pro* ^3^,*D‐Hyp* ^10^]*TL* (compound **132**) [87]. While L‐configured variants generally retained antimicrobial activity comparable to the parent peptide, inversion to the d configuration resulted in a pronounced loss of activity, especially against Gram‐negative bacteria. Notably, incorporation of D‐Hyp significantly reduced α‐helical content, and this loss of structural integrity correlated with decreased antibacterial efficacy, underscoring the importance of maintaining an appropriate helical framework for membrane interaction. An additional strategy to disentangle the role of sequence versus composition has been the use of scrambled analogs. These peptides preserve the overall amino acid composition but alter the spatial distribution of residues. Srivastava et al. designed *TempL SCR‐1* (compound **133**) and *TempL SCR‐2* (compound **134**) by rearranging the hydrophobic residues of TL and its analog **105** [84]. Both peptides exhibited reduced helical propensity and lower aggregation, which translated into markedly diminished antimicrobial and anti‐endotoxin activities, as well as weaker interactions with lipopolysaccharides. A similar trend was observed in scrambled derivatives of other TL analogs. Bellavita et al. generated scrambled versions of peptides **7** and **14** (compounds **135** and **136**), which failed to reproduce the anti‐inflammatory properties of their parent sequences [98].

### 3.5 Emerging Structure–Activity Relationships Landscape

**FIGURE 5:**
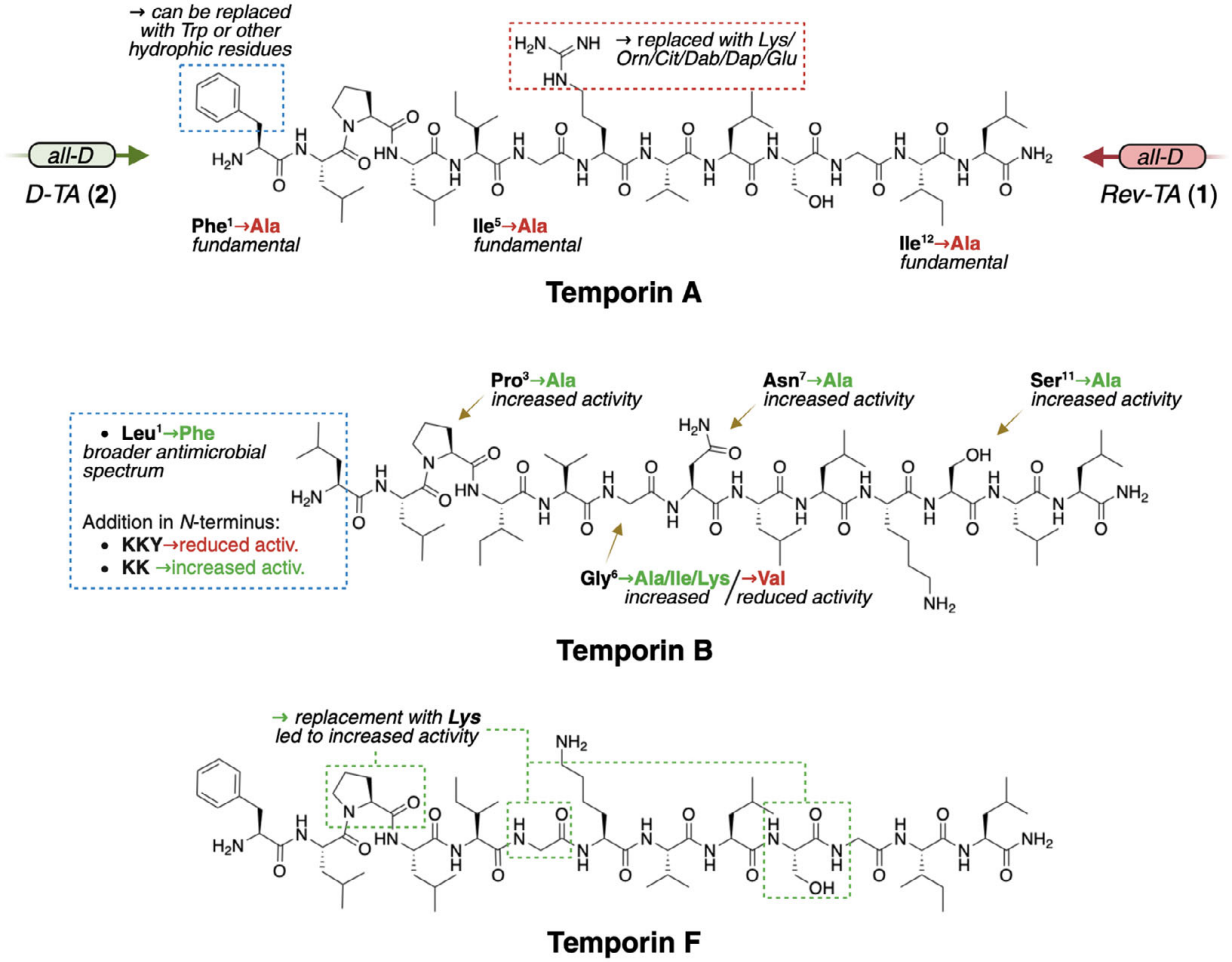
Overview of representative sequence optimization strategies applied to temporins A, B, and F. Amino acid substitutions, stereochemical modifications, and charge modulation identified residues critical for antimicrobial activity and highlighted key SAR trends influencing potency and spectrum of activity.

**FIGURE 6:**
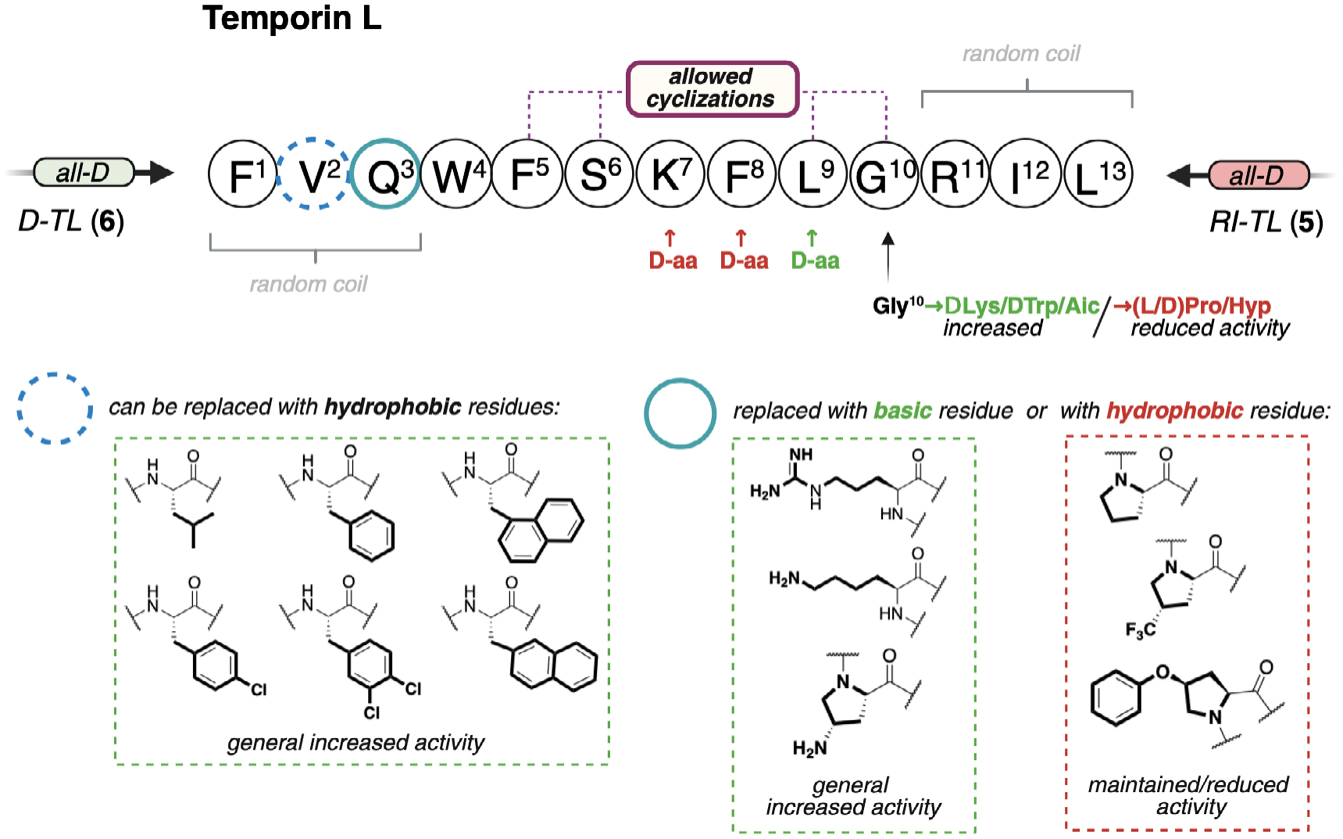
Summary of representative optimization strategies applied to temporin L (TL). Amino acid substitutions, D‐amino acid incorporation, retro‐inverso design, and cyclization approaches were explored to modulate peptide conformation and antimicrobial activity**.**

Collectively, the studies described in the previous sections, focusing on site‐specific modifications, provide a framework of SAR that can guide the rational design of temporin‐based analogs (Figures 5 and 6). Despite differences in sequence and antimicrobial spectrum, TA, TB, and TL share common mechanistic and structural determinants, with antimicrobial activity primarily arising from membrane‐disruptive interactions. Within this context, peptide function emerges from a delicate interplay between backbone stereochemistry, hydrophobicity, charge distribution, and residue positioning, rather than from single dominant features. Importantly, comparative analysis across this temporin family indicates that identical chemical modifications do not necessarily produce equivalent biological outcomes. Instead, their effects are shaped by the intrinsic physicochemical properties of each isoform, emphasizing that successful peptide engineering requires consideration of the structural context in which a given modification is introduced.

One of the most consistent observations concerns the role of backbone stereochemistry. Temporins generally tolerate global stereochemical inversion, as demonstrated by the preserved activity of all‐D analogs of TA and TL [70, 76]. This suggests that their mechanism of action is largely independent of absolute chirality and not based on stereospecific receptor recognition. In contrast, local stereochemical perturbations, such as D‐residue insertions within defined helical regions of both TA and TL, often result in significant loss of activity or altered selectivity [41, 44, 70, 78, 87]. These findings highlight that while the overall fold can be preserved under global inversion, local stereochemical integrity is essential for maintaining the spatial arrangement required for effective membrane interaction. However, comparison across temporin isoforms indicates that the consequences of local stereochemical modifications are context‐dependent. While TA and TL appear particularly sensitive to local stereochemical perturbations within functionally relevant regions, the available evidence for TB indicates that selected D‐amino acid substitutions can be accommodated without substantial loss of antimicrobial activity [77]. A second key determinant is the distribution of charged residues. Across all temporins, antimicrobial activity is not simply dictated by the overall net positive charge, but rather by its precise spatial organization with the amphipathic framework. In TA, systematic substitutions at Arg^7^ demonstrate that preserving both the guanidinium functionality and the native side‐chain length is critical for efficient membrane interaction and antimicrobial activity [53, 70]. Similarly, in TL, increasing net charge does not necessarily enhance potency unless the additional cationic residues are correctly positioned to preserve amphipathicity and membrane selectivity [44, 70, 85, 86, 88, 100]. In TB and TF, charge modulation strategies further confirm that improved antimicrobial activity is achieved not by increasing charge *per se*, but by introducing cationic residues at permissive positions while maintaining the native structural organization. These findings demonstrate that charge optimization relies on the strategic distribution of cationic residues rather than on absolute net charge alone. Hydrophobicity represents another central determinant, acting in concert with charge to define amphipathicity, membrane insertion, and peptide self‐association. Site‐specific modifications in TA, TB and TL consistently show that moderate increases in hydrophobicity, particularly when introduced at permissive positions or combined with appropriate charge compensation, can enhance antimicrobial potency. Conversely, excessive hydrophobic reinforcement or modifications at structurally critical sites frequently promote peptide aggregation, reduce conformational flexibility, increase cytotoxicity, and ultimately impair biological activity. These observations support the existence of an optimal hydrophobicity range that depends on both the parent peptide and the specific site of modification, rather than on hydrophobicity alone. Closely related to hydrophobicity is the contribution of residues defining the membrane‐interacting hydrophobic surface. Alanine‐scanning studies have clearly demonstrated that residues forming this core hydrophobic core, including Phe, Leu, and Ile in TA and the phenylalanine zipper motif in TL, are generally intolerant to substitution, as their alteration compromises both structural organization and antimicrobial activity [70, 78, 84]. In contrast, more permissive positions (e.g., Gly, Ser, or Pro in selected sequence contexts) often tolerate substitutions and can be exploited to fine‐tune activity, helicity, or selectivity. This principle is particularly evident in TB, where only a limited number of positions, most notably Gly^6^, tolerate hydrophobic remodeling, whereas residues directly involved in membrane anchoring remain highly conserved [81, 83, 99]. Noncanonical amino acid substitutions and sequence rearrangements further reinforce the importance of residue positioning and global organization. Scrambled analogs consistently show that preserving amino acid composition alone is insufficient to maintain activity, as disruption of the spatial arrangement leads to loss of function [84, 98]. Similarly, the introduction of noncanonical residues can modulate activity, stability, and toxicity, but their effects are highly context‐dependent, reflecting the tight coupling between local structure and global peptide behavior.

Taken together, these findings define a set of general design principles for temporin analogs: (i) preservation of an amphipathic architecture with a well‐defined hydrophobic face and strategically positioned cationic residues; (ii) maintenance of key residues involved in membrane anchoring, while exploiting tolerant positions for optimization; (iii) fine‐tuning of hydrophobicity within an optimal range to avoid aggregation and toxicity; (iv) careful control of backbone stereochemistry, particularly at functionally critical sites; and (v) consideration of sequence order and conformational adaptability as essential determinants of activity. The emerging SAR landscape highlights that successful optimization of temporin peptides does not rely on maximizing individual physicochemical parameters, but rather on achieving a balanced integration of multiple structural features that collectively govern antimicrobial potency and selectivity.

## 4 Conformationally Constrained TL Analogs

**FIGURE 7:**
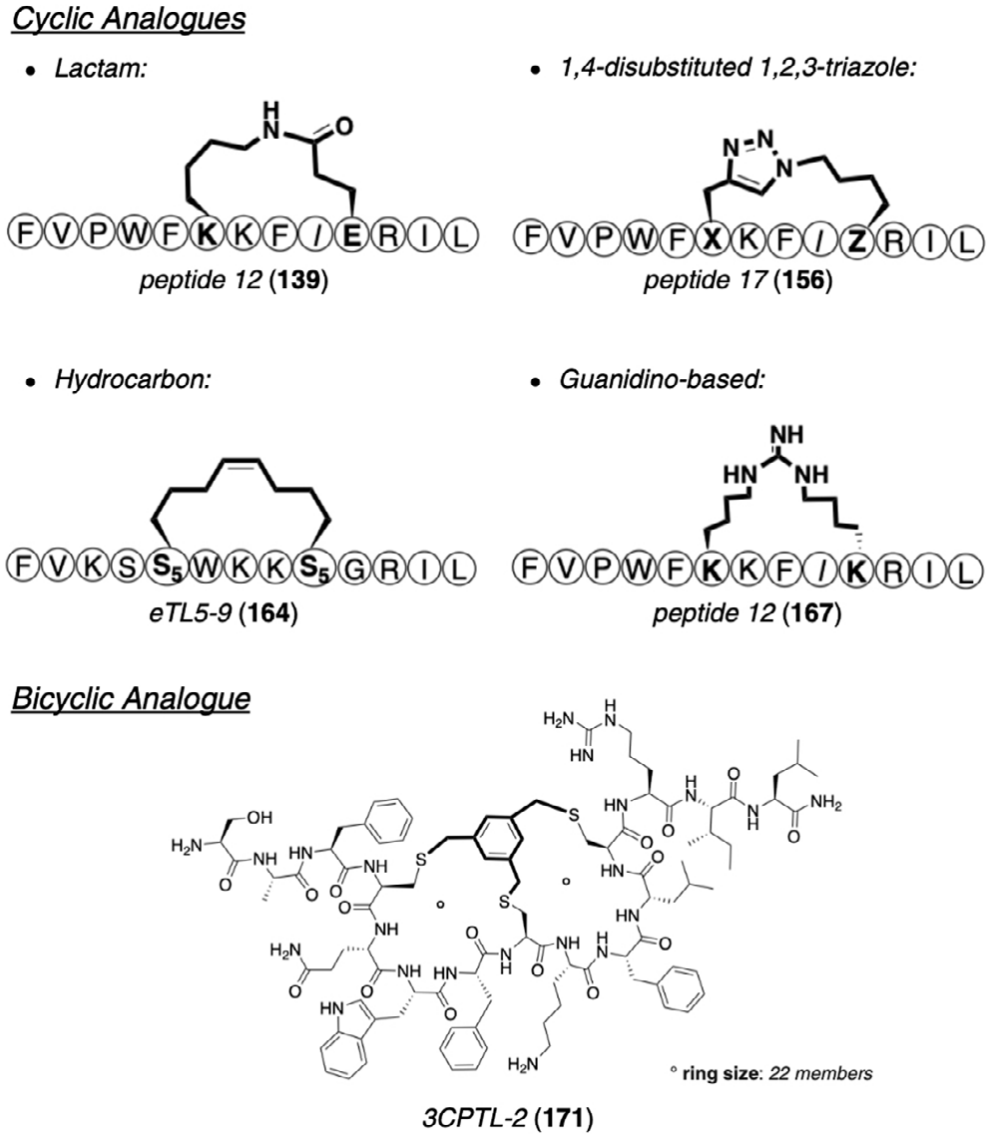
Representative cyclic and bicyclic temporin analogs obtained through different peptide stapling and cyclization strategies.

Cyclization has emerged as a powerful strategy to improve the pharmacological profile of AMPs, as it can enhance proteolytic stability, modulate conformational flexibility, and improve selectivity. By imposing structural constraints, cyclization often promotes and stabilizes bioactive conformations, which are essential for efficient membrane interaction [104, 105]. However, within the *Rana temporaria* temporin family, these approaches have been exclusively applied to TL (Figure 7). In this context, studies on TL consistently demonstrate that both the nature and the position of the conformational constraint are critical in determining biological outcomes.

### 4.1 Lactam‐Based Cyclic Analogs

Starting from peptide **7**, Bellavita et al. developed a series of cyclic derivatives incorporating lactam bridges. In this series, the position of the constraint emerged as a key determinant of activity. In particular, different *i*,*i* + 4 cyclization patterns were explored by introducing Lys/Glu pairs at distinct sequence positions, revealing that the position of the lactam bridge can preserve or improve antimicrobial activity. Among the six cyclic analogs generated (peptides **137–142**), the derivative cyclized between Lys^6^ and Glu^10^ (peptide **139**), displayed the most favorable biological profile. This analog retained the amphipathic α‐helical profile, as confirmed by CD and NMR analyses performed in micellar environments, and displayed enhanced activity against Gram‐positive bacteria, with approximately a 2‐fold reduction in MIC values (from 6.25 to 3.12 μM). Peptide **139** also exhibited antifungal activity (MIC 50 μM against *Candida* spp.) and strong antibiofilm properties (~90% eradication) while showing negligible in vivo toxicity [106, 107]. Further optimization revealed that this system tolerates stereochemical variation. In particular, stereoinversion at Glu^10^ led to derivative **143**, which retained antimicrobial activity comparable to peptide **139**. Moreover, combining lactam cyclization with an increase in cationic character, such as through the introduction of guanidinium as pendant groups, generated analogs **144–151** with enhanced activity, especially against Gram‐negative bacteria (e.g., MIC values against *P. aeruginosa* in the range of 12.5–100 μM) [108]. These findings highlight the synergistic interplay between conformational constraints and charge. In contrast, analogs featuring *i*,*i* + 7 lactam cyclization (peptides **152–153**) displayed markedly reduced activity against both Gram‐positive and Gram‐negative strains (MIC values > 100 μM), underscoring the importance of precise spacing in preserving the functional conformation [106].

### 4.2 Stapled Variants

Alternative cyclization strategies have also been explored using peptide **7** as a scaffold, including disulfide bridges and 1,4‐disubstituted 1,2,3‐triazole linkages. For triazole‐based analogs, different cyclization positions were systematically investigated through both *i*,*i* + 4 and *i*,*i* + 7 constraints (**154–160**), retained activity against Gram‐positive bacteria (compound **156** exhibited MIC values ranging from 1.56 to 3.12 μM), but showed reduced efficacy against Gram‐negative strains (MIC values > 100 μM), likely due to increased self‐aggregation. In contrast, disulfide‐ and hydrocarbon‐bridged analogs (**161–162**) generally exhibited reduced antimicrobial activity [106]. Regarding the impact of these cyclization motifs on secondary structure, CD analysis suggested that peptide **161** did not adopt a well‐defined helical conformation, whereas peptide **162** showed a tendency to form α‐helical aggregates in aqueous solution [106]. Hydrocarbon stapling further highlighted the importance of constraint positioning. Studies on peptide **57** revealed clear position‐dependent effects. Mahto et al. introduced staples at the *N*‐terminal (*eTL1‐5*, compound **163**), central (*eTL5‐9*, compound **164**), and *C*‐terminal regions (*eTL9‐13*, compound **165**) [109]. Stapling within the central region (positions 5–9, compound **164** preserved or improved antibacterial activity (MIC values ranging from 3.6 to 7.2 μM against selected Gram‐negative strains and from 1.9 to 3.6 μM against selected Gram‐positive strains), while maintaining low hemolysis (5.2% at 50–100 μM). In contrast, stapling in *N*‐ and *C*‐terminal regions increased cytotoxicity despite similar antimicrobial effects. Notably, peptide **164** combined improved serum stability with minimal hemolysis, even in the absence of a well‐defined secondary structure, as confirmed by CD analysis. This observation suggests that local conformational constraints, rather than global helicity, may be sufficient to preserve membrane‐disruptive activity [109]. More recently, guanidinium‐based bridges have been introduced as alternative cyclization elements (compounds **166–168**) [108]. These modifications simultaneously increase cationic character while maintaining peptide cyclization, resulting in enhanced antimicrobial activity, particularly against Gram‐negative bacteria (e.g., MIC 1.56 μM against *A. baumannii*), while maintaining low hemolysis and improved proteolytic stability [108].

### 4.3 Bicyclic Analogs

A more advanced level of conformational control has been achieved through bicyclization. In this approach, starting from analog **83** and **84**, bicyclic peptides (compounds **169–172**) using 1,3,5‐tris(bromomethyl)benzene (TBMB) and 1,3,5‐triacryloylhexahydro‐1,3,5‐triazine (TATA) linkers. The introduction of three cysteine residues enabled the formation of two intramolecular bridges, yielding highly constrained architectures. These bicyclic analogs displayed improved potency against the SARS‐CoV‐2 main protease (Mpro), with IC_50_ values in the range of 6–12 μM. In particular, the TBMB‐linked derivative of *TLP‐2* (compound **171**) showed the highest activity (IC_50_ = 6 μM) [110]. These results indicate that bicyclization not only enhances conformational rigidity, as supported by molecular dynamics simulations, but also promotes more effective peptide‐protein interactions favoring persistent and well‐oriented interactions with key catalytic and binding site residues, such as His^41^, Cys^145^, and Glu^166^, within the protease active site, thereby extending the functional scope of temporin‐derived peptides beyond membrane‐targeting mechanisms.

## 5 Summary and Outlook

In this review, we systematically compiled and critically analyzed the structural and point modifications introduced across the *Rana temporaria* temporin A–L family with the aim of identifying general SAR principles that may guide the rational design of next‐generation analogs. Rather than considering individual modifications in isolation, the comparative analysis suggests that antimicrobial activity is influenced by the interplay between backbone stereochemistry, hydrophobicity, charge distribution, residue positioning, and conformational organization. Accordingly, the available studies suggest that successful peptide optimization relies on achieving an appropriate balance among these features within the structural context of each temporin isoform. Despite these advances, several limitations emerge from the current body of literature that hinder a fully rational and comparative interpretation of the available data. One of the most evident issues is the lack of standardization in the reporting of antimicrobial activity. Concentrations are inconsistently expressed, alternating between micromolar units and mass‐based units (e.g., µM and µg/mL), which complicates direct comparison across studies and may obscure meaningful SAR trends. Likewise, differences in cytotoxicity assays, hemolysis protocols, mammalian cell models, and experimental endpoints make it difficult to directly compare the selectivity profiles of different analogs. A more uniform framework for data reporting would therefore greatly enhance reproducibility and facilitate cross‐study analyses. Furthermore, while this review has deliberately focused on sequence modifications, it is important to acknowledge that peptide optimization extends beyond sequence alterations alone. A growing body of work has explored the synergistic use of temporins with conventional antibiotics, as well as their incorporation into advanced delivery systems. These complementary strategies have the potential to improve peptide stability, bioavailability, and overall efficacy and may therefore represents valuable components of future optimization efforts. Finally, the available evidence remains largely based on in vitro antimicrobial, hemolytic, and cytotoxicity assays, while more comprehensive biological evaluation remains comparatively limited. Antimicrobial activity is often assessed against different bacterial strains, making it difficult to draw consistent conclusions regarding spectrum and potency. Moreover, not all derivatives have been systematically evaluated across multiple biological activities, such as antifungal or anti‐inflammatory effects, resulting in a fragmented understanding of their full therapeutic potential. Importantly, only a limited subset of the most promising and active derivatives has been further validated against clinical isolates and/or in vivo infection models, which represents a crucial step toward assessing their translational relevance. Establishing more standardized panels of microbial strains, including clinically relevant isolates, and expanding the scope of biological assays, ideally extending to in vivo validation for the most active compounds, would contribute to a clearer and more comprehensive characterization of these peptides.

In conclusion, the studies discussed in this review suggest that the biological performance of temporins is governed by the balanced integration of multiple structural determinants rather than by any single physicochemical parameter. The emerging SAR landscape indicates that the effects of stereochemistry, hydrophobicity, charge modulation, and residue positioning are intrinsically context‐dependent and must therefore be interpreted within the framework of each individual peptide scaffold. Future efforts should prioritize standardization, broader biological evaluation, and integration of complementary strategies beyond sequence design. Such advancements will be essential to fully harness the potential of this peptide family and to enable the rational development of temporin‐derived antimicrobial agents with improved efficacy and translational potential.

## Conflicts of Interest

The authors declare no conflicts of interest.

## Data Availability

Data sharing not applicable to this article as no datasets were generated or analysed during the current study.
